# Rate after-effects fail to transfer cross-modally: Evidence for distributed sensory timing mechanisms

**DOI:** 10.1038/s41598-018-19218-z

**Published:** 2018-01-17

**Authors:** Aysha Motala, James Heron, Paul V. McGraw, Neil W. Roach, David Whitaker

**Affiliations:** 1 School of Optometry and Vision Sciences, Cardiff University, Cardiff, CF24 4HQ United Kingdom; 2 Bradford School of Optometry and Vision Science, University of Bradford, Bradford, BD7 1DP United Kingdom; 3 Visual Neuroscience Group, School of Psychology, The University of Nottingham, Nottingham, NG7 2RD United Kingdom

## Abstract

Accurate time perception is critical for a number of human behaviours, such as understanding speech and the appreciation of music. However, it remains unresolved whether sensory time perception is mediated by a central timing component regulating all senses, or by a set of distributed mechanisms, each dedicated to a single sensory modality and operating in a largely independent manner. To address this issue, we conducted a range of unimodal and cross-modal rate adaptation experiments, in order to establish the degree of specificity of classical after-effects of sensory adaptation. Adapting to a fast rate of sensory stimulation typically makes a moderate rate appear slower (repulsive after-effect), and vice versa. A central timing hypothesis predicts general transfer of adaptation effects across modalities, whilst distributed mechanisms predict a high degree of sensory selectivity. Rate perception was quantified by a method of temporal reproduction across all combinations of visual, auditory and tactile senses. Robust repulsive after-effects were observed in all unimodal rate conditions, but were not observed for any cross-modal pairings. Our results show that sensory timing abilities are adaptable but, crucially, that this change is modality-specific - an outcome that is consistent with a distributed sensory timing hypothesis.

## Introduction

In recent decades many attempts have been made to understand the processes underlying timing judgements. Notable theoretical developments include proposals for internal clock mechanisms^1,2^ that manage converting objective time into subjective time^3^. At the heart of the internal clock model sits the presence of an internal regulator or ‘pacemaker’ mechanism that emits a steady series of pulses. The number of pulses emitted during a particular time window are then counted by an ‘accumulator’ which subsequently determines temporal duration^2^. On the other hand, the channels hypothesis suggests that, analogous to visual modules of motion and orientation, distinct subcortical channels exist dedicated to processing specific features of time^4,5^. Efforts to deconstruct the components of subjective time have used duration judgements of intervals^6,7^, comparing timing abilities across senses with cross-modal interval discrimination^8,9^ and assessing the accuracy and flexibility of timing judgements by varying sensory presentation through induced asynchrony adaptation^10^.

Fundamentally, it is not yet clear whether sensory timing mechanisms operate on a centralised and supramodal basis with one, generalised timing faculty regulating timing across sensory systems or, rather, that distributed mechanisms exist with multiple internal clocks overlooking each individual sense^11–14^. Evidence from rate perception experiments, show that concurrently presented auditory stimuli bias judgements of visual flicker, suggesting mechanisms exist that allow the shared communication (and influence) of temporal information across the senses; consistent with a centralised rate processing mechanism^15,16^. Conversely, in studies of duration adaptation, it has been found that after repeated presentations of specific durations, contingent after-effects were found across unimodal conditions, yet the same effects were absent cross-modally. These results are at odds with the centralised theory of timing and instead suggest the presence of distinct and distributed mechanisms, each separately modulating the perception of time^17^.

The judgement of temporal rate is one area that has produced substantial disagreement in previous literature. Becker & Rasmussen^18^ demonstrated strong repulsive after-effects on a test beat, following adaptation to a train of beats of varying temporal frequency. Adapting to a fast train of beats made a moderate train appear slower, and vice versa. Crucially, however, adapting to an auditory beat had no effect on a subsequent test train of visual flashes – in other words, the after-effect was limited to the sensory modality of the adapting stimulus. A very different outcome was reported in a more recent study by Levitan and colleagues^19^. After being exposed to a 5 Hz temporal frequency presentation, a 4 Hz presentation was perceived as slower than its physical rate. Again, the effect occurs bi-directionally in the sense that after being exposed to a 3 Hz temporal frequency modulation, the same 4 Hz test stimulus then appeared much faster. Critically, Levitan and colleagues reported that adaptation-induced after-effects transferred across modalities (bi-directionally in audition and vision). This result is somewhat surprising as temporal frequency perception is generally thought to be a low-level process operating during the earlier stages of sensory analysis, perhaps as early as receptor surfaces. Thus, a temporal frequency after-effect that transfers across modalities implies that these early sensory pathways are also cross-modal in nature. Controversy over the existence, or otherwise, of cross-modal adaptation after-effects can also be found in other studies of sensory timing^15,20–22^. Disagreement over such a fundamental issue of centralised versus distributed timing mechanisms significantly limits our progress in developing models of how humans quantify sensory time. It is currently unclear whether we should promote a supramodal, central timing mechanism or a series of timing mechanisms co-existing and operating similarly to one another in different senses^23^.

Here we address this issue by mapping out the magnitude and temporal extent of rate adaptation effects across the auditory and visual systems and extend these findings to the previously unexplored tactile modality. In particular, we ask whether adaptation in one sense carries over to representation in a different sensory modality, using a method of rate reproduction.

## Results

Mean reproduction and corresponding mean standard error values were calculated for each adapting temporal frequency for each sensory combination. A best-fitting curve was fitted to the data representing the first derivative of a Gaussian, as in Heron *et al*.^5^ to extract relevant parameters such as the magnitude and extent of any adaptation effects.

**Figure 1:**
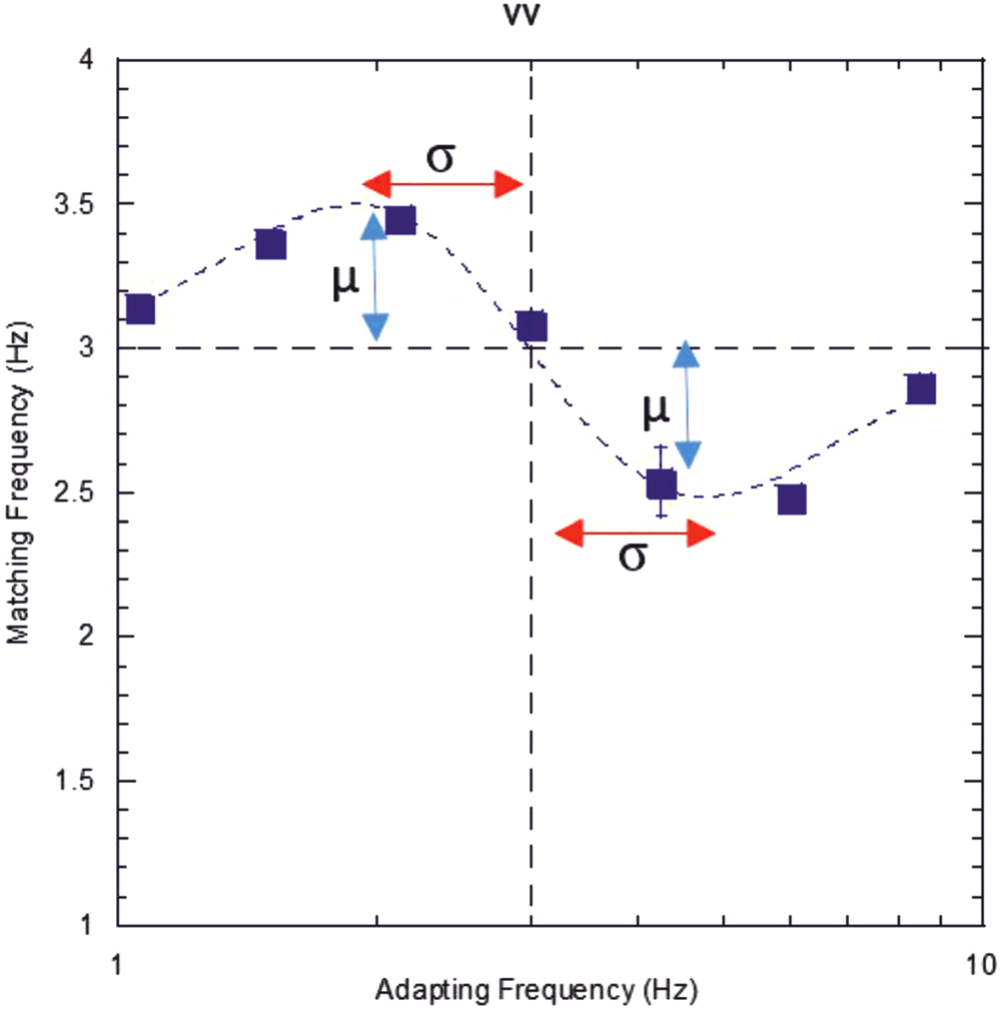
Example data for the unimodal visual condition. The subject was exposed to a range of adapting temporal frequencies in the visual modality and was required to match the perceived rate of a subsequent 3 Hz test stimulus, also in the visual modality. The curve fit to the data represents the optimum (least-squared residuals) fit of Equation 1. Vertical blue arrows indicate the amplitude of the effect (µ) and the horizontal red arrows indicate the spread of effect (σ), error bars indicate standard error.

Figure 1 shows this function fitted to a data set from one observer, in a condition where the adapting and test stimuli were of the same modality (both visual). The data demonstrate that adapting to a slower rate than 3 Hz, results in a 3 Hz test stimulus appearing faster than it actually is (close to 3.5 Hz for this observer), whereas adapting to a faster rate than 3 Hz subsequently makes the same 3 Hz presentation feel significantly slower than it actually is; a bi-directional after-effect. This adaptive shift is band-limited, in that the matching frequency tends to return to baseline levels (or at least level off in magnitude) for large temporal differences between adaptor and test. This pattern of results is entirely consistent with the rate after-effects presented by Becker and Rasmussen^18^, and is also typical of other classic repulsive after-effects that result from visual adaptation^24–27^.

**Figure 2:**
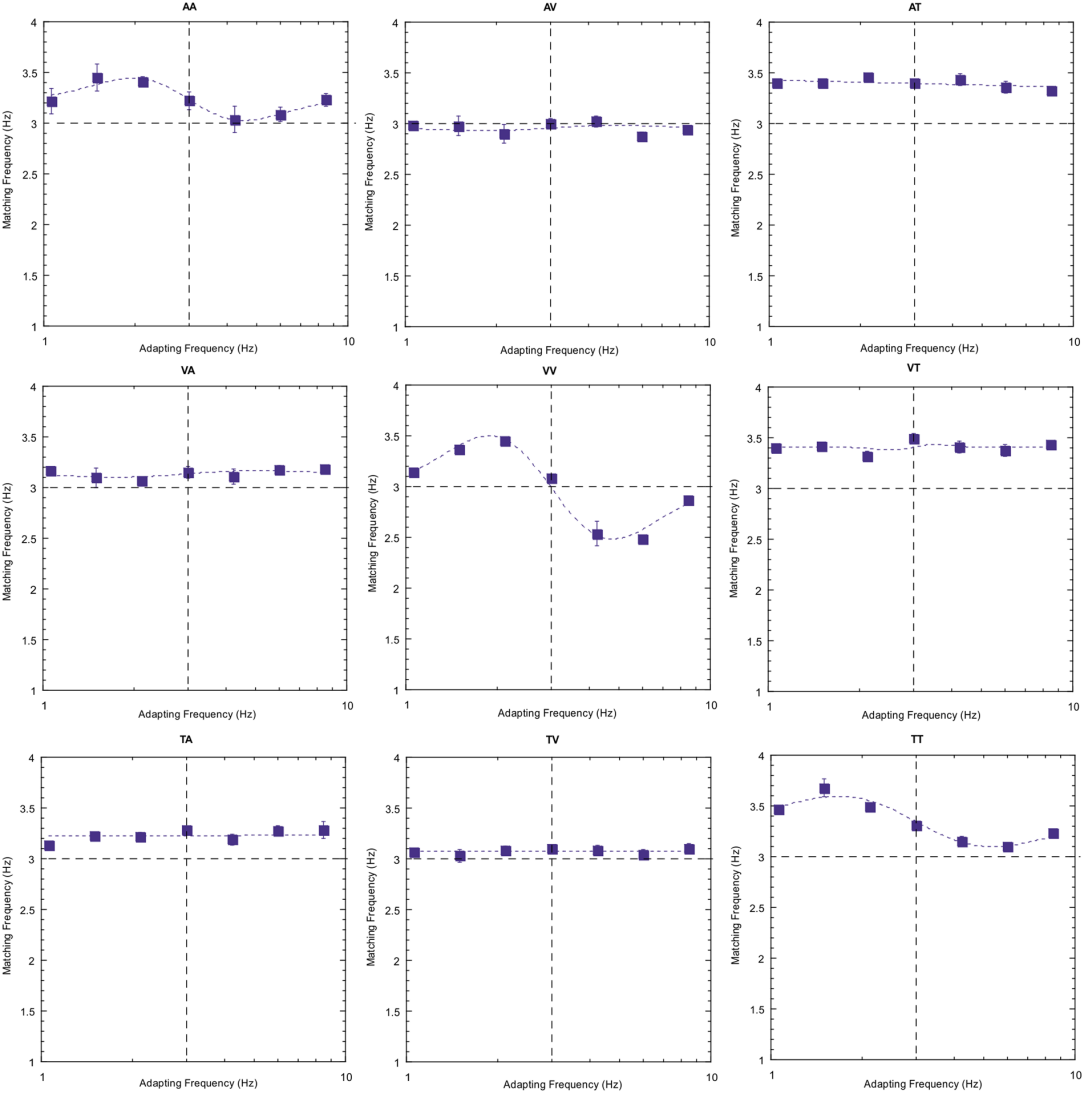
Data for all nine adapt/test stimulus pairings for subject DW. The sensory combination is shown at the top of each plot and follows the key presented previously. The three unimodal conditions are shown diagonally from top left to bottom right. Error bars indicate standard error. See text for further description.

Data for this same subject are now plotted for all nine possible adapt/test combinations of the three stimulus modalities (Table 1, Fig. 2). The key to the sensory combinations is shown in the following table (adapting modality followed by test modality; ‘A’ denotes auditory, ‘T’ denotes tactile and ‘V’ visual).

**Table 1:** Schematic displaying all possible modality combinations that were tested. The first letter denotes the adapting modality and the second letter denotes the testing modality.

**AA**	**AV**	**AT**
**VA**	**VV**	**VT**
**TA**	**TV**	**TT**

**Figure 3:**
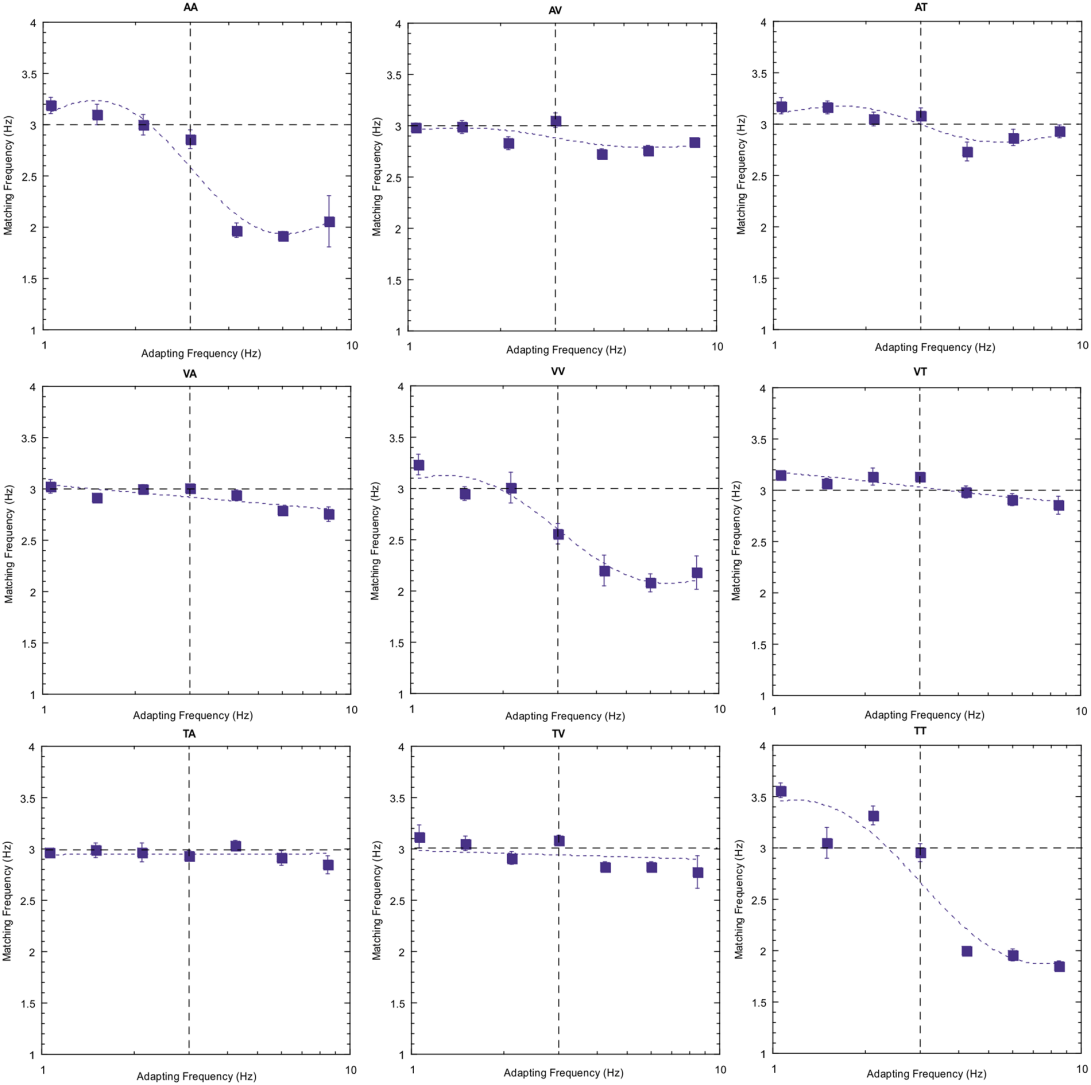
As for Fig. 2 but for subject AM.

**Figure 4:**
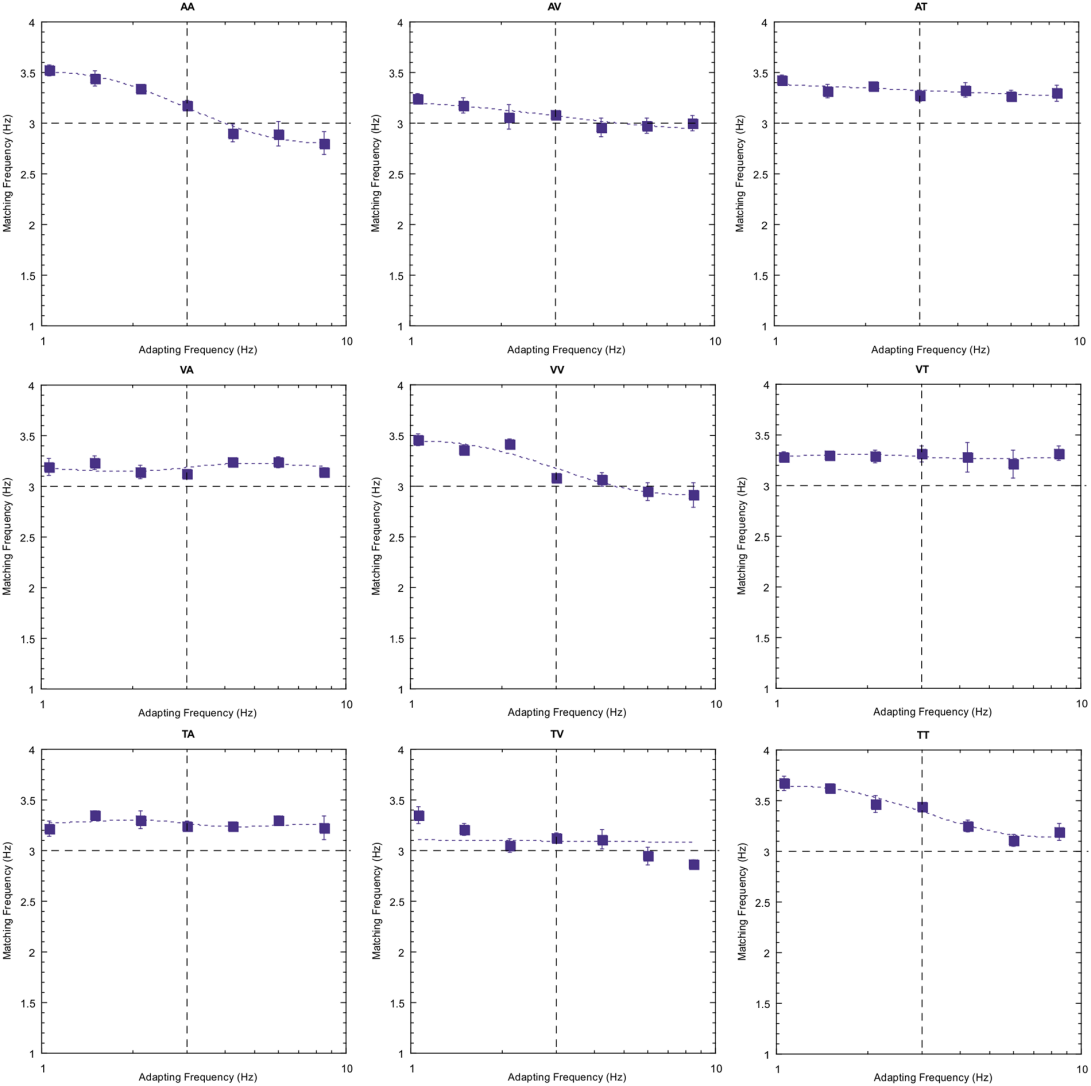
As for Fig. 2 but for subject YL.

Note first that the reproduced rates of the 3 Hz test stimulus are not consistently veridical. This particular subject tends to overestimate rate for the tactile stimuli (right hand column) and, to a lesser extent, the auditory test stimuli (left hand column). This type of individual variation in perceived rate across sensory domains been reported previously^28,29^. Of more interest, however, is the modulation in perceived rate as a temporal rate difference is introduced between the adapting and test stimuli. Two clear effects emerge; first, robust repulsive after-effects are found for each of the unisensory conditions. Second, for all other conditions (the cross-modal conditions) there are no systematic variations in the magnitude of adaptation effects across the adapt/test stimulus range. Equivalent plots for two other observers are also presented below (Figs 3 and 4) and show a very similar pattern of results.

The effect size of the adaptation effect (μ) and temporal extent (spread) of the adaptation effects (σ) are shown in Table 2 for each of the unimodal conditions. Subject AM shows the largest adaptation effects, averaging 0.66 Hz (22% of baseline temporal frequency). No clear pattern emerges of a consistent difference in amplitude of effect size across the three sensory conditions. Subject DW shows the tightest tuning of effect size, with the largest temporal spread of effect shown by the naïve observer YL. Again, no clear pattern of any inter-sensory differences in spread of effect is evident. The significance of effect sizes for all nine sensory combinations were calculated by dividing the amplitude (μ) value by its standard error. A two-tailed, one-sample t-test (df = 6) was then conducted for each subject in each condition and a further Holm-Bonferroni correction was conducted for all subjects to determine the significance of adaptation effects. All unimodal conditions show highly significant effect sizes (p < 0.01), but this is not the case for any cross-modal pairings (p > 0.05) (Table 2).

**Table 2:** Amplitudes of adaptation effect (μ), spread (σ in log units) of adaptation effect and Holm-Bonferroni adjusted p-values across all unimodal conditions for each subject. Error values represent standard error.

	Subject AM			Subject DW			Subject YL		
	Amplitude (μ)	p-value	Spread (σ)	Amplitude (μ)	p-value	Spread (σ)	Amplitude (μ)	p-value	Spread (σ)
AA	0.65 ± 0.09	0.002	0.31 ± 0.06	0.21 ± 0.03	<0.001	0.18 ± 0.02	0.35 ± 0.03	<0.001	0.45 ± 0.09
TT	0.8 ± 0.14	0.010	0.40 ± 0.15	0.25 ± 0.03	<0.001	0.25 ± 0.03	0.25 ± 0.03	0.002	0.43 ± 0.12
VV	0.53 ± 0.06	<0.001	0.36 ± 0.08	0.51 ± 0.04	<0.001	0.21 ± 0.01	0.26 ± 0.05	0.009	0.43 ± 0.16

A potential problem with the data presented thus far is that observers were always aware of the test modality to be presented, and which they subsequently were required to reproduce. There is the possibility that observers somehow failed to attend to the adapting stimulus if they were aware that the test stimulus was going to be in a different modality. Becker and Rasmussen^18^ acknowledged this possibility, whilst considering it unlikely to have affected their findings. Levitan *et al*.^19^ went a step further, and actively controlled attention to the adapting stimulus by the use of a practical gap-counting paradigm during the adaptation phase. We therefore ran a control experiment using the auditory/visual pairing in which the modality of the test stimulus was unknown to the observer – 50% of trials were auditory, the other 50% were visual. The paradigm is a simple one – any purposeful attentional strategy during the adaptation phase would affect both auditory and visual test stimuli alike, meaning that any adaptation effects should either be present or absent in both test conditions. Alternatively, should adaptation persist in the unimodal condition yet remain absent in the cross-modal pairing, then the potentially contaminating role of attention during the adaptation phase can be eliminated.

**Figure 5:**
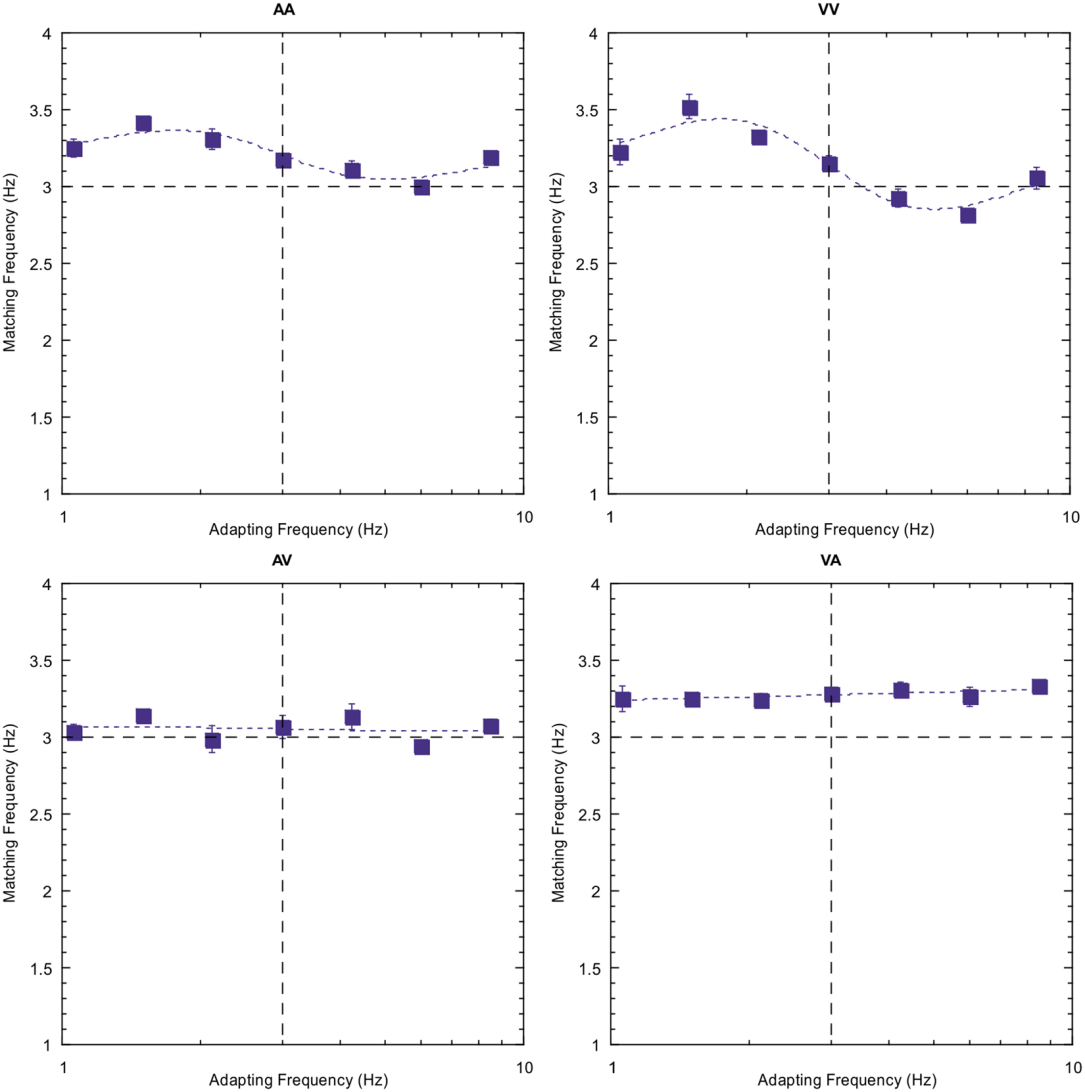
Data from the control experiment using the auditory/visual pairing (subject DW). Left-hand plots represent the auditory adaptation condition, right-hand plots visual adaptation. Upper plots represent unimodal conditions (adapt and test same modality), lower plots cross-modal conditions; error bars indicate standard error. See text for a description of the control methodology.

Results are shown in Fig. 5 for subject DW (for other subjects see Supplementary Figures S1 and S2 and Supplementary Table S1). The findings are conclusive – marked after-effects occur for both adaptation conditions (auditory, left column; visual, right column) only when the test stimulus is of the same modality (upper plots), but not when they are a different modality (lower figures).

## Discussion

Our findings reveal strong band-limited repulsive after-effects for rate perception in all three sensory systems of audition, touch and vision. However, rate perception is unchanged when the modality of the adapting stimulus differs from that of the test. Adaptation is thought to result from the sensory history of neural populations; when the adapting and test stimuli activate overlapping populations, the effects on perception are revealed as repulsive after-effects. Thus, evidence that rate after-effects fail to transfer across sensory modalities suggests that rate adaptation occurs relatively early in the sensory processing hierarchy, perhaps in the sensory cortices themselves. There is physiological evidence for temporal frequency-selective neurons within the respective cortices^30,31^ and these may well form the basis for temporal ‘channels’^32,33^. The processing of temporal rate therefore appears to share a strong commonality with other channel-based temporal judgments such as perceived duration^5,17^ despite suggested differences in neural substrates^34^.

Neural evidence for ‘tuned’ temporal representations in humans was recently presented by Hayashi *et al*.^35^ using an fMRI duration adaptation paradigm. When a subject was repeatedly presented with stimuli of the same duration, a substantially decreased level of activity was reported in the right inferior parietal lobule (IPL). Testing on a range of subsecond durations produced the same result, suggesting neurons in the human IPL are preferentially tuned to specific subsecond durations^35^. Studies of perceptual learning in the auditory domain also show that learning effects are ‘tuned’ to the temporal interval that has been trained^36,37^. Similar temporally-specific practice effects are found in somatosensory interval discrimination^38,39^. These findings suggest a dedicated circuitry underlying the processing of specific timeframes, reinforcing the band-limited tuning found in the current rate adaptation experiments.

**Figure 6:**
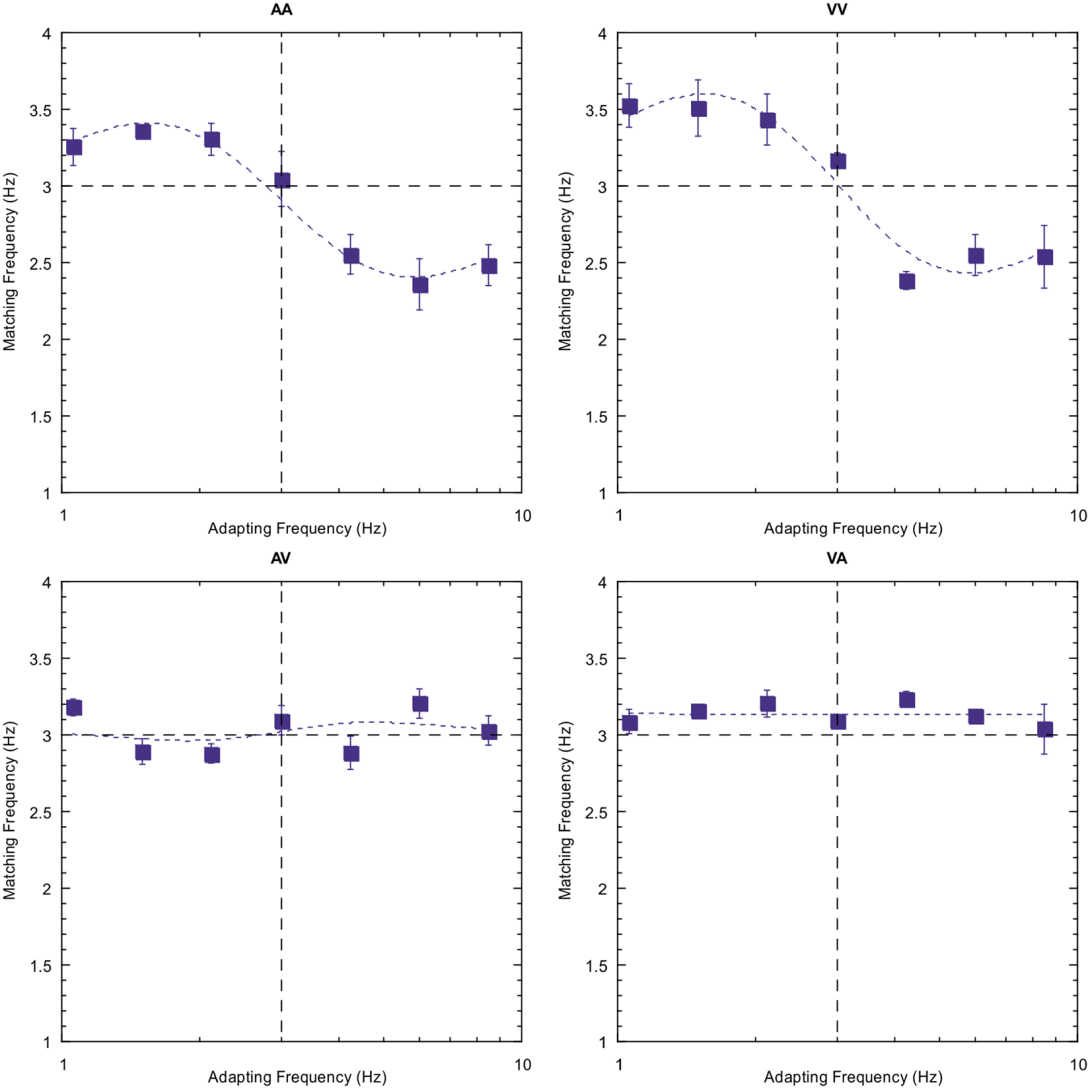
Data for all four adapt/test stimulus pairings for subject AM where stimuli were spatially and temporally overlapped. The sensory combination is shown at the top of each plot. The two unimodal conditions are shown in the top panel (left; AA, right; VV) whereas the cross-modal conditions are presented in the lower panel (left; AV, right; VA). Error bars indicate standard error. See text for further description.

There is some evidence to suggest that the processing of sensory time is spatially-specific^38,40^. In addition, some multisensory interactions are extinguished when component unisensory stimuli are presented at sufficiently disparate spatial locations^41^. It may be, therefore, that after-effects are only present when the spatial locations of sensory stimuli overlap. Our main experiments presented auditory stimuli over headphones and visual stimuli on a display – perhaps this is the reason behind our inability to find cross-modal adaptation effects? To address this potential criticism, we conducted a further control experiment in which auditory and visual stimuli were spatially coincident. This was achieved by projecting visual stimuli onto a thin fabric sheet allowing transparency of acoustic signals, and simultaneously projecting auditory stimuli via a speaker placed directly behind the screen. All other features of the set-up were kept consistent with our main experiment. Data was gathered for all possible pairings encompassing the auditory and visual modalities (AA, VV, AV, VA) over a minimum of 105 trials for each observer. Sample data from one observer is presented below (Fig. 6) and plots from another observer and data from all statistical tests are in supplementary materials (Supplementary Figure S3 and Supplementary Table S2):

Results clearly follow the same pattern of findings as our main and control experiments: adapting to a given rate in the adapting phase significantly affects the perception of a test rate but only when both adapting and test stimuli belong to the same sensory modality. Our findings indicate that, whilst some temporal aftereffects become manifest only when adapting and test stimuli are co-localised in space, this is not sufficient to produce any cross-modal effects in sensory rate adaptation.

Our data are consistent with those of Becker and Rasmussen^18^ even when extended to a new sensory modality (tactile) and show that temporal rate perception is highly specific to sensory modality. The question arises as to why Levitan *et al*.^19^ found significant cross-modal effects in rate perception? Their experimental approach was to use a method of single stimuli in which the frequency of a test sequence was compared with an internal mean, generated through an initial testing phase. This is a very efficient method, yet it has the much-criticised disadvantage^42^ that the internal mean can be readily corrupted by subsequent stimulus presentation. Levitan and colleagues employed a lengthy period of rate adaptation, yet, they also employed a lengthy period of test stimulus presentation and no ‘top-up’ adaptation thereby potentially neglecting the role of decay in adaptation after-effects or corruption of the internal mean by the test stimuli themselves. However, inconsistent with this explanation is the fact that their effects, like ours, were tuned. A 4 Hz signal in one sense was distorted by a 5 Hz adaptor in another, yet it was less affected by an 8 Hz adaptor, and not at all by 12 Hz. This suggests that their effects were genuine sensory distortions resulting from adaptation rather than higher-level distortions in an internal cognitive representation.

Some studies suggest the existence of both low-level, modality-specific timing mechanisms and higher-level modality-independent processes. Stauffer *et al*.^43^ modelled the accuracy of rhythm perception in different senses and found their data were best described by a two-stage model. Further support for the existence of a common, amodal timing mechanism comes from the duration literature^44^. Additionally, results from interval timing suggest a level of interaction between independent auditory and visual processing systems^45^. It is reported that when subjects are instructed to compare standards of continuously presented and flickering visual stimuli, whilst ignoring simultaneously presented auditory flutters, auditory flutters influence the subjective judgements of visual stimuli. Results suggest that whilst interval timing perception is largely governed by modality-dependent mechanisms, interactions between auditory and visual modalities can, on occasion, modulate perception^45^. The very fact that we are able to compare temporal rate in one sense with that in another indicates that some higher-level analysis of multisensory rate must exist, but our data indicate that this comparison stage is not susceptible to cross-modal rate adaptation. Levitan’s data however, seem to suggest that these after-effects are bandwidth-limited for both low-level modality-independent processes and also for their higher-level, modality-independent counterparts.

Our perceptual system appears to deal with sensory input on a dynamic basis. Interestingly, when sensory stimuli are presented concurrently, temporal information from the two senses interferes with one another in subsequent rate discrimination tasks^46^ yet when the same sensory stimuli are presented successively, as they were in the present tasks, rate perception appears to remain segregated by sensory modality. Thus, the structure of sensory presentation holds influence over how segregated temporal processing will be, suggesting that adaptation mechanisms precede multisensory integration.

In summary, using a sensory adaptation paradigm we demonstrate that our senses are rapidly susceptible to adaptation effects in the perception of temporal rate, but only when adaptation and test are of the same modality. This supports the existence of distributed timing mechanisms, each specific to a particular sensory modality. Future work might examine whether similar adaptation effects occur in the perception of single intervals (gaps between brief sensory pairs). This would tell us whether there is something special about ‘rate’ or whether it is simply equivalent to a train of sensory ‘gaps’.

## Methods

### Subjects

3 participants (2 female and 1 male) (mean age = 33) participated, with self-reported normal hearing and visual abilities. Following initial practice sessions, a lengthy process (20–25 hours) of data collection began, in a series of sessions spread over several weeks. Two of the participants (authors) had previous experience of psychophysical data collection. The third participant had no such experience and was naïve to the purpose of the experiments. The experiments received ethical approval from the Research Ethics Committee at the School of Optometry and Vision Sciences, University of Cardiff and all experiments were performed in accordance with relevant guidelines and regulations. Informed consent was obtained for study participation.

### Stimuli

Brief (16 msec duration) sensory stimuli were presented – either in the auditory, visual or somatosensory modality and all stimuli were grossly suprathreshold. Stimulus generation and presentation was controlled by an Intel ® Core ™ i5–4460 desktop computer running Microsoft Windows 7. The programming environment involved MATLAB 8.6 (Mathworks, USA) in combination with Psychophysics Toolbox 3 (http://www.psychtoolbox.org). Stimulus timing was verified using a dual-channel oscilloscope.

### Visual

Visual stimuli were presented on an Eizo EV2436W monitor. These were bright (274 cd/m^2^) white circular flashes presented centrally against a uniform dark background (0.32 cd/m^2^). Stimulus duration was a single frame (approximately 16ms at the monitor frame rate of 60 Hz). At the viewing distance of 60 cm the circular flash subtended a diameter of approximately 10.5 degrees of visual angle.

### Auditory

Auditory stimuli consisted of brief (16ms duration) bursts of white noise generated by a Xonar Essence STX (ASUS) soundcard (https://www.asus.com/us/Sound-Cards/Xonar_Essence_STX/) with a sampling rate of 44,100 Hz. Stimuli were delivered using Sennheiser HD 280 Pro Headphones at an SPL of 70 dB. Auditory stimuli for the second control experiment where auditory and visual stimuli arose from the same spatial location were delivered using a TEAC two-way speaker system (http://www.teac-audio.eu/en/products/ls-101hr-129505.html).

### Tactile

Tactile stimuli were produced using the amplified (LP-2020A + Lepai Tripath Class-T Hi-Fi Audio Mini Amplifier) ‘audio-out’ voltage of the sound card which controlled a miniature electromagnetic solenoid-type stimulator (Dancer Design Tactor http://www.dancerdesign.co.uk/products/tactor.html). Using brief (16ms) audio bursts of white noise the Tactor produced taps to the index finger of the left hand. The Tactor was enclosed within a fabric occluder in order to eliminate the possibility of auditory cues.

### Responses

Subjects were required to reproduce a given sensory rate by tapping with their right forefinger on a piezoelectric transducer (https://www.amazon.co.uk/Piezo-electric-disk-transducer-15mm/dp/B01K8X9E5K). The resulting voltage output was fed to the ‘audio in’ of the soundcard as a recording which was analysed within MATLAB to extract the average temporal frequency of tapping. The transducer was enclosed in a sound-dampening environment and shielded from sight of the subject. To further eliminate the possibility of auditory feedback, white noise was played via the headphones throughout the tapping response phase.

### Procedure

**Figure 7:**
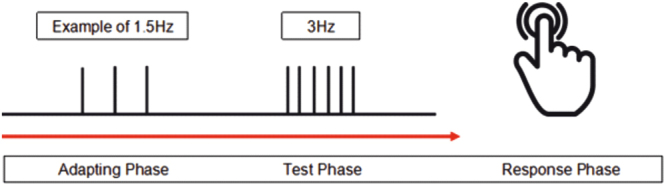
Schematic demonstrating the three phases of each trial (see text for details). Response phase drawing accessed from www.iconsmind.com.

A single trial began with an adaptation period (8–10 seconds) of a pre-chosen temporal frequency (ranging between 1.06 Hz and 8.46 Hz in seven logarithmically-spaced steps). This was followed by a test phase (lasting between 2 and 2.5 seconds) of 3 Hz stimulation, after which the subjects were required to reproduce the perceived rate of the test phase by tapping the piezoelectric transducer for approximately 5 seconds. Subjects knew to start responding when they heard the white-noise mask begin (to eliminate a possible auditory cue from tapping) (Fig. 7).

After a total of 5 trials the mean of the tapping rate in the response phase was presented on the computer monitor and recorded. A break of at least 3 minutes was then taken before another run with a different adapting frequency and adapt/test pairing – this was to ensure no adaptation effects crossed-over from one run to the next. Each adapting temporal frequency for each adapt/test pairing was repeated 5 times in total to give a mean and standard error of the mean. The order of testing conditions was randomised. Additionally, a control experiment was conducted using the audio-visual pairing, in which the modality of the test phase was unknown to the observer on any trial.

The datasets generated and analysed during the current study are available from the corresponding author on reasonable request.

## Electronic supplementary material

Supplementary Information
