# Supplementary material for: Rate after-effects fail to transfer cross-modally: Evidence for distributed sensory timing mechanisms
Source: Sci Rep. 2018 Jan 17;8:924. doi: 10.1038/s41598-018-19218-z (PMC5772423; doi:10.1038/s41598-018-19218-z)
Supplement: Supplementary file 1 — Supplementary Information [file 41598_2018_19218_MOESM1_ESM.pdf]

Rate after-effects fail to transfer cross-modally:

Evidence for distributed sensory timing mechanisms – Supplementary Information

Aysha Motala[1], James Heron[2], Paul V. McGraw[3], Neil W. Roach[3] and David Whitaker[1]*

[1] School of Optometry and Vision Sciences, Cardiff University, Cardiff CF24 4HQ, United Kingdom [2] Bradford School of Optometry and Vision Science, University of Bradford, Bradford BD7 1DP, United Kingdom [3] Visual Neuroscience Group, School of Psychology, The University of Nottingham, Nottingham NG7 2RD, United Kingdom

*Correspondence should be addressed to David Whitaker (whitakerd@cardiff.ac.uk)

Figure S1. As for figure 5 but for subject AM.

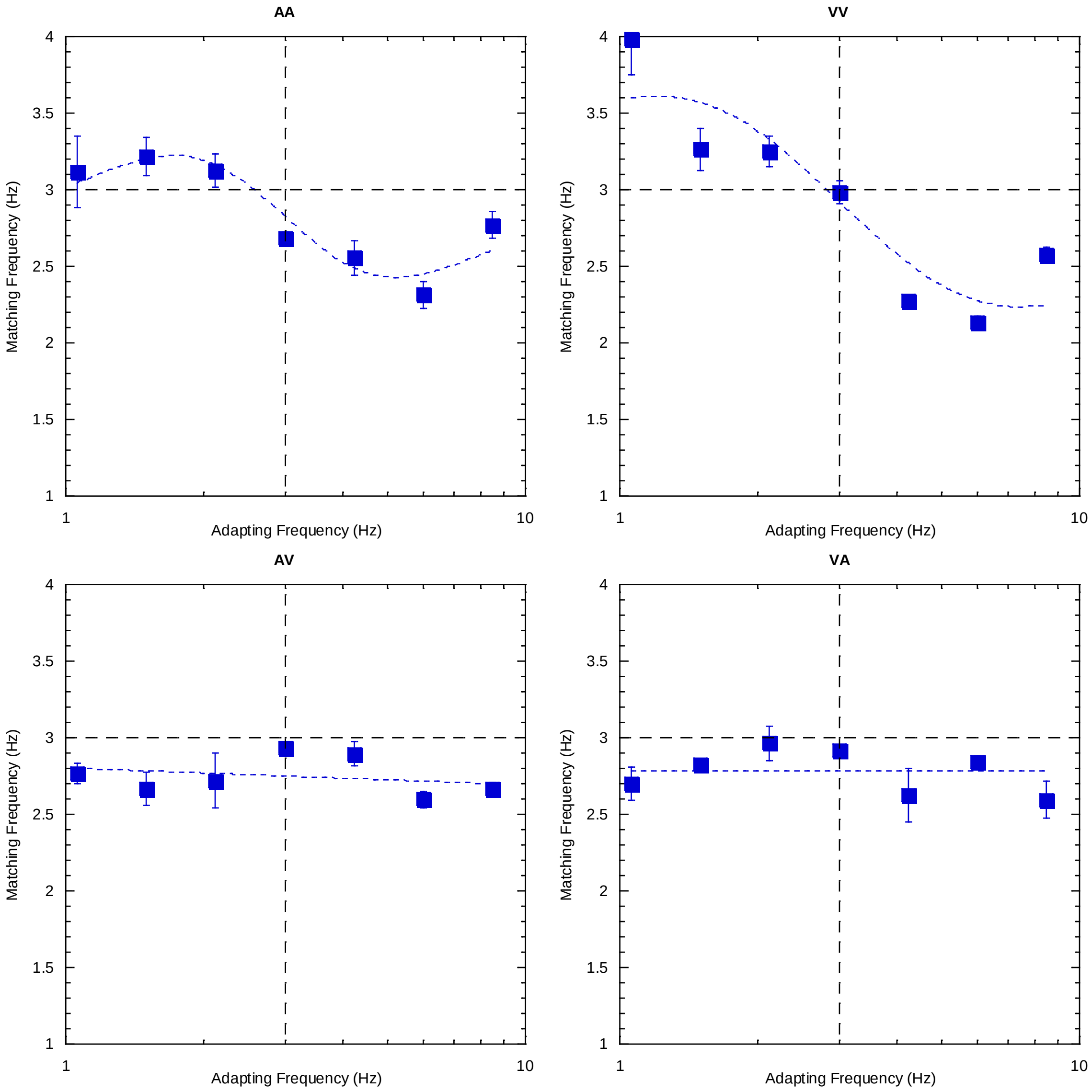

Figure S2: As for figure 5 but for subject YL.

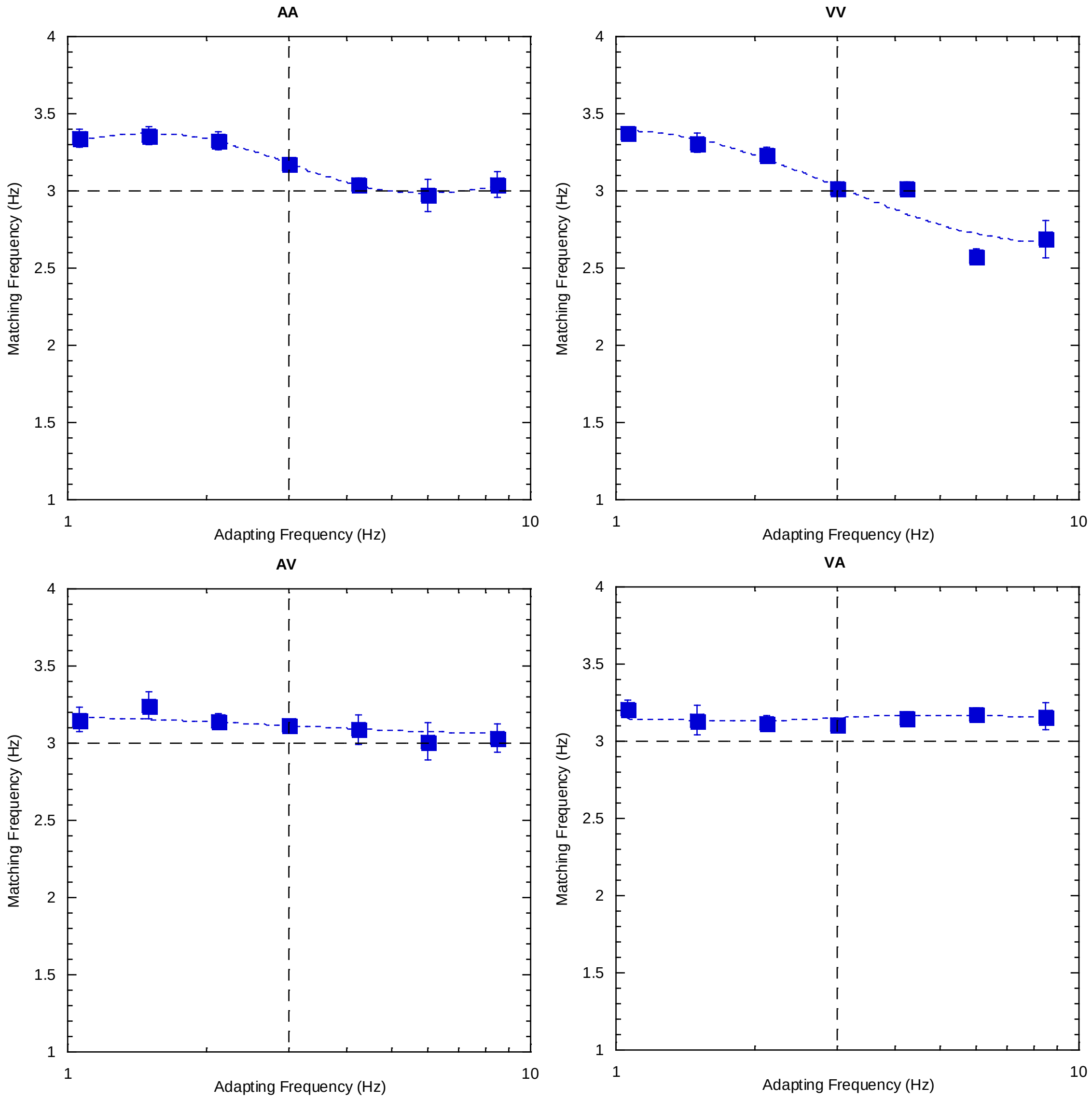

Figure S3: Data for all four adapt/test stimulus pairings for subject DW where stimuli were spatially and temporally overlapped. The sensory combination is shown at the top of each plot. The two unimodal conditions are shown in the top panel (left; AA, right; VV). Error bars indicate standard error. See text for further description.

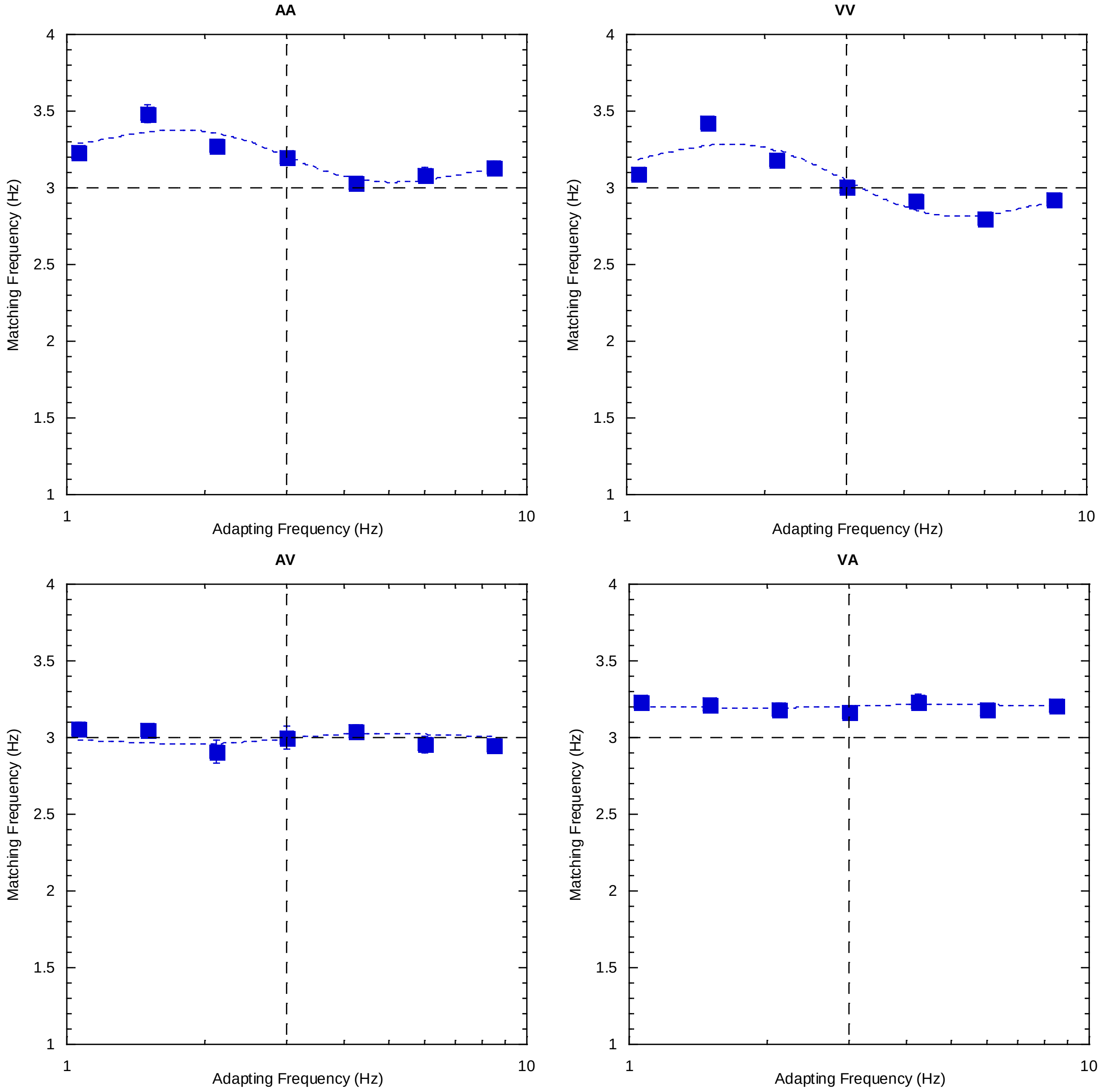

Table S1: Amplitudes of adaptation effect (μ), spread (σ in log units) of adaptation effect and Holm-Bonferroni adjusted p-values across all unimodal conditions for each subject for the control experiment in which subjects were unaware of the test modality. The first letter denotes the adapting modality and the second letter denotes the testing modality, 'A' refers to the auditory modality and 'V' to the visual modality. All cross-modal conditions were found to be not statistically significant (p>0.05).

	Subject AM			Subject DW			Subject YL		
	**Amplitude (μ)**	**p-value**	**Spread (σ)**	**Amplitude (μ)**	**p-value**	**Spread (σ)**	**Amplitude (μ)**	**p-value**	**Spread (σ)**
**AA**	0.40±.08	0.007	0.24±.04	0.16±.04	0.014	0.23±.05	0.19±.01	<0.001	0.29±.01
**VV**	0.69±.17	0.020	0.39±.20	0.29±.05	0.003	0.23±.03	0.36±.10	0.036	0.49±.27

(Error values represent standard error)

Table S2: Amplitudes of adaptation effect (μ), spread (σ in log units) of adaptation effect and Holm-Bonferroni adjusted p-values across all unimodal conditions for each subject for the control experiment in which visual and auditory stimuli were spatially and temporally overlapped. The first letter denotes the adapting modality and the second letter denotes the testing modality, 'A' refers to the auditory modality and 'V' to the visual modality. All cross-modal conditions were found to be not statistically significant (p>0.05).

	Subject AM			Subject DW		
	**Amplitude (μ)**	**p-value**	**Spread (σ)**	**Amplitude (μ)**	**p-value**	**Spread (σ)**
**AA**	0.50±.04	<0.001	0.29±.03	0.17±.05	0.030	0.23±.05
**VV**	0.58±.08	<0.001	0.29±.04	0.24±.06	0.022	0.25±.06

(Error values represent standard error)
